# Mutations in Lettuce Improvement

**DOI:** 10.1155/2011/723518

**Published:** 2012-01-11

**Authors:** Beiquan Mou

**Affiliations:** Agricultural Research Service, United States Department of Agriculture (USDA), 1636 East Alisal Street, Salinas, CA 93905, USA

## Abstract

Lettuce is a major vegetable in western countries. Mutations generated genetic variations and played an important role in the domestication of the crop. Many traits derived from natural and induced mutations, such as dwarfing, early flowering, male sterility, and chlorophyll deficiency, are useful in physiological and genetic studies. Mutants were also used to develop new lettuce products including miniature and herbicide-tolerant cultivars. Mutant analysis was critical in lettuce genomic studies including identification and cloning of disease-resistance genes. Mutagenesis combined with genomic technology may provide powerful tools for the discovery of novel gene alleles. In addition to radiation and chemical mutagens, unconventional approaches such as tissue or protoplast culture, transposable elements, and space flights have been utilized to generate mutants in lettuce. Since mutation breeding is considered nontransgenic, it is more acceptable to consumers and will be explored more in the future for lettuce improvement.

## 1. Introduction

There is an increasing demand by consumers for nutritious foods that improve physical performance, reduce risks of diseases, and increase the life span. Lettuce (*Lactuca sativa *L.), commonly found in salad mixtures and sandwiches, is an important component in western diet and nutrition. Lettuce was the second most consumed fresh vegetable in the USA at 28.0 pounds per capita in 2008, behind potato at 36.7 pounds [1].

 Lettuce belongs to the family Asteraceae (Compositae), tribe Cichorieae. Of about 100 species of *Lactuca*, only three (*L*.* serriola* L., *L*.* saligna *L., and *L*.* virosa *L.) can be crossed to lettuce by conventional hybridization methods and thus form the most important breeding group. They are all self-fertilized diploids with 2*n* = 2*x* = 18 chromosomes. There are five major types of lettuce: crisphead (iceberg), romaine, leaf, butterhead, and stem. Stem lettuce is mainly produced in China. In 2010, 58% of the lettuce production in the USA was of the head type, 29% was romaine, and 13% was leaf types [2].

 Genetic variation is very important for any crop breeding program. The inbred nature of lettuce dictates the relatively limited genetic variability in the crop as compared to cross-pollinated crops. Mutation is a valuable tool to create novel traits for lettuce plants and can be classified as natural mutations and induced mutations (Table 1). Natural mutations are still occurring in the crop and its wild relatives, though at a low rate, and resulting beneficial characters can be selected for human needs. Mutagenic agents, such as X-rays, ultraviolet radiation, neutrons, protons, alpha, beta, and gamma rays, or chemical mutagens like ethyl methane sulfonate (EMS), are used to either increase mutation rates or yield mutants unavailable from natural sources. This paper provides a review of the role mutation has played in the evolution and improvement of lettuce.

## 2. Domestication of Lettuce

Lettuce grew in the wild prior to domestication by humans. It is still not exactly clear which species were involved in the evolution that led to today's lettuce. However, it is certain that one of or the only direct ancestor(s) is *L. serriola* [3–6]. *L. sativa* and *L. serriola* cross freely with each other, and the chromosomes of the two species are very similar morphologically [7]. The two taxa are considered by some as subspecies of the same species. It is most likely that changes in *L. serriola* caused by mutations led to the appearance of favorable traits that were liked by humans, particularly forms without spines on stems and leaves and with large seeds. They were then selected for propagation and further modified to fit human needs. These early forms could have been suitable for animal use or for oil from the seeds for domestic consumption. Several primitive forms still exist and are used for these purposes in Egypt today [8, 9]. Most of these grow and develop rapidly and have non-reflexed involucres to prevent seed shattering, large seeds, and high oil content in the seeds (35%, [10]). For example, one of these landraces, known as the USDA Plant Introduction (PI) 251245 from Egypt, is used for seed oil.

Domestication of the wild types of lettuce has led to the loss of prickles from leaves and stems, less latex and tissue bitterness, reduced suckering, slow bolting except for stem lettuce, and increased seed size. Human selection and later breeding efforts have also resulted in changes in size, shape, color, texture, and taste of leaves and plants, heading habits, resistance to diseases and insects, yield, and adaptation to different geographic areas and environments.

## 3. New Genes for Growth and Development 

 Lettuce typically grows as a rosette-type plant with an array of leaves arranged in a whorl during the vegetative growth phase. When plant enters reproductive stage, bolting, or initiation of rapid stem elongation, occurs. Premature bolting can cause loss or deformation of heading, which renders a commercial lettuce crop unacceptable. Waycott et al. [11] used EMS treatment of lettuce seeds to generate several dwarf mutants that were found to be controlled by four dwarfing loci. In comparison to the wild-type, the recessive lettuce dwarfs had reduced stature, shortened internodes, darker green leaves, and modified flower morphology. Dwarf mutants have lost their ability either to produce active gibberellic acid (GA) or to respond to active GA that is involved in regulating lettuce stem elongation [12]. These mutants could be used in a breeding program for resistance to premature bolting.

 Lettuce flowers in >100 days when grown under mostly long days. A single mutant plant was selected from a crisphead breeding line, USDA 56679. It bolted while still in the seedling stage, bypassing the rosette and head formation stages, and flowered about 55 days from planting [13]. Six genes that control flowering time in lettuce have been reported [14]. Early flowering genes can be used to speed up generation time in genetic and growth studies. In breeding, a modified backcross procedure has been developed in which a desired gene can be coupled with a dominant early-flowering gene and transferred to a recurrent parent in half the time required for normal lettuce [15]. The early alleles are easily eliminated at the end of the cycle by inbreeding. This method was used to transfer a lettuce mosaic resistance gene to the cultivars ‘Prizehead' and ‘Salinas' [16].

 A male-sterile mutant was found in the crisphead cultivar ‘Calmar' [17]. Seven male sterile alleles have been identified in lettuce [18], which has potential use for creating F_1_ hybrids. Although hybrid vigor has been observed in some crosses, as a self-pollinated species, seed companies are likely to be more interested in the protection of the hybrid lettuce cultivars. However, hybrid seeds have not been used in commercial lettuce production, mainly due to two reasons as follows. (1) None of the male sterility genes have a cytoplasmic factor to make possible entirely male sterile populations for seed production, so a commercial hybridization program would not be cost effective. (2) Lettuce pollen is sticky and not windblown, and there is a dearth of pollinating insects for lettuce flowers. Another way to create hybrid cultivars is multiplication of a single hybrid plant by tissue culture. But that may be cost prohibitive, runs the risk of genetic variation, and needs to change the current direct seeding to a transplant system in the USA lettuce production.

 A common form of induced plant mutation is chlorophyll deficiency. Haque and Godward [19] found that 26% of M_1_ plants (plants grown from treated seeds) exhibited chlorophyll mutations and 24% had stem fasciations after ^60^Co irradiation of dry *Lactuca *and *Cichorium *seeds, with *L. serriola *showing greater sensitivity to radiation than *L. sativa*. Seven recessive chlorophyll deficient mutant alleles have been described in lettuce [20]. As an abnormality, these mutants are less likely to be useful in a breeding program. However, they may contribute to lettuce genetic map, potential markers of other genes, and physiological or nutritional studies.

## 4. Miniature Lettuce 

 An early flowering and dwarf double-mutant 86–1024 was developed from EMS-treated lettuce seeds. 86–1024 was then crossed to a crisphead cultivar ‘Salinas,' and miniature plant selections were made in the progeny, leading to the release of three minicrisphead cultivars: ‘Ice Cube,' ‘Blush,' and ‘Mini-Green' [21]. They closely resemble standard cultivars in appearance, but attain about one-half the diameter (8 to 12 cm). A minilettuce head can be consumed by a person in one meal, giving consumers more choices. Now you can find minilettuce products in certain high-end supermarkets and restaurants in the USA.

## 5. Herbicide Tolerance 

 Weeds in leafy vegetables can reduce yields, quality, and harvest efficiency. Herbicides registered for leafy greens in the USA are few and old, and these are in jeopardy due to low sales and high cost of regulatory compliance. American lettuce growers have depended on pronamide (Kerb) as the major herbicide. In 2009 the US Environmental Protection Agency stopped the use of Kerb on leaf lettuce because of crop reclassifications. There is no certainty that Kerb will be permitted to use on leaf lettuce again, as the herbicide has been classified as a carcinogen and found in ground water. The seedling and foliage of lettuce are very sensitive to herbicide damage; therefore, attempts to find new herbicides have not succeeded. Herbicide-tolerant crop is an attractive alternative, since growers can simply spray their fields with a broad-spectrum herbicide that removes weeds without injuring the tolerant crop.

 A naturally occurring mutant of sulfonylurea herbicide-tolerance was identified in *L. serriola* from a northern Idaho field in 1987 [22]. The mutant has a proline 173 to histidine substitution in Domain A of the acetolactate synthase (ALS) enzyme, a region demonstrated to play a pivotal role in conferring resistance to herbicides that inhibit ALS [23]. The specific activity of ALS from the mutant was 57% less than the specific activity of ALS from the wild type, suggesting that the mutation may affect enzyme function, expression, or stability [24]. In addition to sulfonylurea, the mutant also was cross-resistant to several other ALS-inhibitor herbicides including representatives of the imidazolinone (imazethapyr) and triazolopyrimidine (flumetsulam) families [24]. The tolerance is inherited as a single incompletely dominant gene [25]. The trait was later transferred into a butterhead cultivar ‘Bibb' through backcrosses [26].

 EMS, gamma ray, and fast neutron were used to treat seeds of lettuce cultivars ‘Diana' and ‘Saffier' in an attempt to find mutants with tolerance to carfentrazone, imazamox, rimsulfuron, glufosinate, and glyphosate herbicides [27]. Yet no progeny plant showed high levels of tolerance.

## 6. Resistance to Downy Mildew 

With the completion of the sequencing projects for human, *Arabidopsis thaliana*, and other model organisms, we have entered the genomic era. Genomic research may impact lettuce breeding by providing a better understanding of the structural organization and functional properties of genes and genome, allowing efficient selection with molecular markers and high-throughput genotyping technologies, and increasing the gene pool available by supplying new sources of desired traits, germplasm, or transgenes. Much of the genomic studies in lettuce have concentrated on resistance to diseases, particularly downy mildew. Downy mildew is caused by a fungus *Bremia lactucae* Regel and is favored by relatively low temperatures and high humidity. Dominant genes in lettuce known as *Dm *genes match the avirulence (Avr) genes in the pathogen in a gene-for-gene fashion and confer race-specific immunity to seedlings as well as adult plants [28–31]. So far, over 20 *Dm *or *R *(resistance factor) genes have been identified. Conserved motifs within sequences of the over 20 cloned plant disease resistance genes from a variety of plant species, such as putative nucleotide binding sites, have allowed the isolation of resistance gene candidates (*RGC*) from numerous plant species using polymerase chain reaction (PCR) with degenerate primers [32]. Several multigene families of *RGC* sequences have been identified in lettuce, one of which was clustered at the *Dm3 *locus (the *RGC2 *family) [33].

 A variety of mutagens have been used to produce mutations at several *Dm *loci in lettuce. A panel of fast-neutron- (FN-) generated *Dm *mutants was utilized to identify and map molecular markers closely linked to *Dm *genes including *Dm3* [34, 35]. All these mutants had only a single specificity altered to a completely susceptible phenotype, and each genetic lesion mapped to an individual *Dm *locus. Several of these mutants did not have flanking molecular markers and were, therefore, due to deletions at the respective *Dm *loci [34–36].

 Mutants generated in many plant species are being used increasingly in the identification and cloning of genes. Allelism tests of the lettuce *dm3* mutants indicated that *Dm3 *specificity was encoded by a single gene [37]. A member of the *RGC2* family, *RGC2B*, was identified as a potential candidate for *Dm3 *based on genetic and molecular analysis of *dm3 *mutants. *RGC2B*-specific markers were missing in a panel of nine *dm3 *deletion mutants [36, 37]. Sequencing analysis of six EMS-induced *dm3 *mutants revealed a variety of point mutations in *RGC2B*, and losses of resistance were due to single changes in amino acid sequence or a change in an intron splice site [32]. A full-length genomic copy of *RGC2B *was isolated from a lambda-phage library and introduced into a cultivar lacking *Dm3 *specificity and an EMS-induced *dm3* mutant. Transgenic expression of *RGC2B *in these genotypes resulted in their resistance to all isolates expressing *Avr3 *from a wide range of geographical origins [32]. The transgenic complementation proved that *RGC2B *encodes *Dm3 *resistance. The mutant analyses and transgenic experiments demonstrated that the *RGC2B *gene was both necessary and sufficient to provide *Dm3*-encoded resistance. 

 The *Dm3* gene is approximately 14.5 kb long with seven introns and a 5.9 kb transcript [32]. A 703 bp intron in the 5′-untranslated region places the promoter at least 731 bp upstream of the initiation codon. It has an unusually large leucine-rich repeat- (LRR-) encoding region that is about twice the size of LRR-encoding regions of resistance genes found in other plant species. Two mutants with deletions in the C-terminal LRR demonstrate that this region is required for *Dm3 *activity and is consistent with the spontaneous loss of *Dm3 *activity due to a gene conversion event in this region [38]. The frequency of *Dm3 *in natural populations of *L. serriola *is very low (one in 1033 accessions analyzed) due to deletions and frequent gene conversions at the *RGC2 *locus [39]. Unequal crossover, insertion/deletion, and point mutation events contributed to the evolution of this locus [40].

## 7. Lettuce Improvement by TILLING 

 A reverse genetic method called TILLING (targeting induced local lesions in genomes) uses chemical mutagenesis followed by screening for single-base changes to discover novel alleles [41]. With TILLING, PCR is used to amplify a targeted region of the genome from DNA samples of thousands of mutated plants. The PCR product is heated and reannealed to form heteroduplexes between mutated and wild-type DNA. Heteroduplexes are cleaved at mismatched sites by the *Cel *I endonuclease, and cleavage products can be visualized by size separation from the full-length PCR product on a polyacrylamide gel. DNA from the positive plant is sequenced to reveal the exact nature of the mutation. Using this approach, large populations can be screened rapidly to obtain numerous point mutations in any targeted gene. To identify genes underlying important traits for sustainability and product quality, a TILLING population of four thousand individuals from the lettuce cultivar ‘Saladin' was developed and DNA as well as seed samples were collected [42]. TILLING has also been used to increase the shelf life of lettuce plants (Claire McCollum, personal communications).

## 8. Unconventional Ways to Generate Mutants 

 Most lettuce mutants have been derived from spontaneous mutations, ionizing radiation (e.g., gamma rays or neutrons), or chemical mutagenesis (e.g., EMS treatment). Anderson et al. [35] found that the frequency of irradiation-induced mutations of *Dm3* gene (9/2211) was significantly higher than the frequency of spontaneous mutations (2/3400). However, other unconventional approaches have been attempted to generate mutations in lettuce.

 Plants regenerated from tissue or protoplast culture are often genetically dissimilar from the source plants, and such changes are termed “somaclonal variation.” Heritable variation has been reported in lettuce plants that were derived from callus [43] and protoplast cultures [44, 45]. Engler and Grogan [45] found that 10 out of 119 protoplast-derived clones segregated for mutant phenotypes (altered cotyledon, leaf, or plant color and/or shape) in approximately 3 : 1 ratios suggesting single-gene recessive mutations. They also identified a variant with increased seedling vigor, a potentially useful characteristic. Some mutations arisen from tissue or protoplast culture may be cytoplasmically inherited [43, 45].

 The maize transposable elements *Activator*/*Dissociation *(*Ac*/*Ds*) were used to develop a transposon mutagenesis system in lettuce for cloning *Dm *genes. Experiments with transposon tagging utilizing the *Ac *and *Ds *generated recessive transgenic mutants in which a dwarf phenotype with abnormal root and shoot development and the loss of *Dm3 *specificity cosegregated with the T-DNAs [37, 46]. However, the low frequency of *Ac *transposition in lettuce [47] determined that map-based cloning was a more successful approach to cloning *Dm3 *gene. The tobacco (*Nicotiana tabacum*) element Tnt1, one of the few identified active retrotransposons in plants, was used successfully for gene tagging in lettuce [48]. Eight out of ten transgenic plants contained at least 28 transposed copies of Tnt1, often inserted into genes. Tnt1 insertions were stable in the progeny of the primary transformants, some of which showed phenotypic alterations due to recessive mutations. One of these mutations was due to Tnt1 insertion in the gibberellin 3*β*-hydroxylase gene.

 Space flights with cosmic radiation and microgravity environment have been utilized to generate mutations for genetic improvement in many crops including lettuce. Space flights resulted in decreased germination rate and chromosomal aberrations of lettuce seeds [49, 50]. Although chromosomal changes happened in lettuce seeds both exposed and not exposed to cosmic particles during space flights, the frequency of these changes were higher in the exposed seeds [51]. The frequency of such aberrations increases with the duration of the flight, a fourfold increase of mutagenesis in lettuce seeds with regard to both the total number of aberrant cells as well as the formation of single cells with multiple aberrations was observed after prolonged exposure (up to 175 days) to space flight factors including cosmic rays [52]. Interestingly, a recessive lettuce mutant at a single locus, *weary*(*wry*), exhibits reduced gravitropic response in hypocotyls and inflorescence stems, although roots still display normal gravitropism. Compared to wild-type hypocotyls and inflorescence stems that had amyloplasts in a single layer of endodermal cells, *weary* hypocotyls contained amyloplasts in several layers of cortical cells, and *weary *inflorescence stem cells lacked amyloplasts entirely [53]. These results are consistent with the proposed role of sedimenting amyloplasts in shoot gravitropism of higher plants [54]. Due to its suitability for hydroponic culture, lettuce is considered by the National Aeronautics and Space Administration to be a priority crop for use in advanced life support systems under conditions of microgravity, such as space stations [55]. Studies of *weary *mutant help understand the interactions between gravitropism and essential plant developmental processes and will undoubtedly allow better prediction and control of plant growth and productivity in microgravity.

## 9. Perspectives 

 Mutations can make profound impact on the evolution and improvement of a self-pollinated crop such as lettuce. It is primarily a means of creating genetic variability, which can then be utilized in physiological or genetic studies and cultivar development. It is most effective to alter qualitative traits under the control of major genes such as disease resistance, development, and quality, rather than quantitative traits like yield and adaptation. Therefore, mutagenesis is particularly suitable for lettuce improvement as disease resistance and quality traits are usually more important than yield traits in vegetable crops. It can create novel traits, while conventional cross-breeding usually just recombines traits that already exist in parents. Since it generally alters only a single or a few genes, the desirable traits can stabilize and breed true in a relatively short period of time through several generations of inbreeding. It may not need a lot investment in equipment and training, especially with chemical mutagenesis. As demonstrated in Section 6, mutants are tremendously useful in genomic studies and gene cloning. Combined with genomic advances and new technologies like TILLING, mutagenesis is becoming an even more powerful tool for lettuce breeders. Since it is considered nontransgenic, mutation breeding is more acceptable to consumers and faces little opposition from the environmentalists, governments, growers, and the general public.

In spite of the many advantages of mutation breeding, this technology has not been fully exploited in lettuce. Breeders may not have access to the equipment for ionizing radiation such as a nuclear reactor or other specialized radiation sources. Most chemical mutagens are carcinogenic. The fear for these chemicals and the lack of ability to manage the waste from mutagen treatments prevent a wider adoption of this useful tool in lettuce breeding programs. Although the mutagenesis process may not be complicated, it still takes some practice and experience to get the right mutagen dosage that delivers high mutation frequency but low mortality rate. Due to the random nature of mutagenesis, a large population of mutated individuals needs to be established to be practically useful. That requires input of great amount of time, labor, facility, and effort. Since most mutations are recessive, screening or selection of mutants is generally not practiced in the M_1 _generation. It may take a large greenhouse space to self the M_1_ plants and generate enough seeds for selection in the M_2_ generation. It would be very cost effective if such a mutant population is established with seeds maintained in a public gene bank, and the lettuce breeding and research community can request seeds to screen for various beneficial traits. Another problem is that mutants are generally not included in the lettuce germplasm collections around the world. They are scattered in the hands of individual researchers and are in danger of being lost. These precious resources should be categorized and preserved like other lettuce cultivars and PI lines in seed gene banks. Despite these issues, mutations have been and will continue to be an integrated part of lettuce improvement. Mutagens have been employed for some 90 years to modify plants for human needs, and their use will only be explored by more and more lettuce researchers in the future.

## Figures and Tables

**Table 1:** Phenotype and genotype of major lettuce mutants derived from natural and artificial sources.

Function	Source	Trait	Gene	Reference
Domestication of lettuce	Natural	Loss of prickles from leaves and stems, less latex and tissue bitterness, reduced suckering, slow bolting, reduced seed shattering, and increased seed size and oil content		[3–6]
Growth and development	EMS	Dwarfs with reduced stature, shortened internodes, and loss of ability to produce or respond to GA	*dwf-1*, *dwf-2, dwf-3, dwf-4 *	[11, 12]
	Natural	Early flowering	*Ef-1, ef-2, Ef-3, ef-4, Ef-5, Ef-6*	[13–16]
	Natural	Male sterility	*ms-1, ms-2, ms-3, ms-4, Ms-5, ms-6, Ms-7 *	[17, 18]
	Natural, EMS	Chlorophyll deficiency	*cd-1, cd-2, cd-3, cd-4, cd-5, cd-6, cd-7*	[19, 20]
	Natural	Reduced gravitropic response in hypocotyls and inflorescence stems	*weary*	[53]
Miniature lettuce	EMS	About one-half the diameter of normal crisphead lettuce	Derived from crosses with *dwf-1 *mutant	[21]
Herbicide tolerance	Natural	Tolerance to sulfonylurea, imidazolinone, and triazolopyrimidine	A single incompletely dominant gene	[22–26]
Downy mildew resistance	Natural, *γ* ray, fast neutron, EMS, transposon	Complete loss of resistance specificities	*dm1, dm3, dm7, dm5/8*	[32, 34–40]
